# Supplementary material for: The impact of phosphorus on projected Sub-Saharan Africa food security futures
Source: Nat Commun. 2022 Oct 29;13:6471. doi: 10.1038/s41467-022-33900-x (PMC9617890; doi:10.1038/s41467-022-33900-x)
Supplement: Supplementary file 1 — Supplementary Information [file 41467_2022_33900_MOESM1_ESM.docx]

**The impact of phosphorus on projected Sub-Saharan Africa food security futures**

**Supplementary Information**

Daniel Magnone^1^*, Vahid J. Niasar^2^*, Alexander F. Bouwman^3,4^, Arthur H. W. Beusen^3,4^, Sjoerd E.A.T.M. van der Zee^5^ and Sheida Z. Sattari^6^

1 University of Lincoln (UK); 2 University of Manchester (UK); 3 Utrecht University (Netherlands); 4 PBL Netherlands Environmental Assessment Agency (Netherlands); 5 Wageningen University (Netherlands); and 6 Ag-Space Ltd (UK).

*Corresponding authors: [dmagnone@lincoln.ac.uk](mailto:dmagnone@lincoln.ac.uk) & [vahid.niasar@manchester.ac.uk](mailto:vahid.niasar@manchester.ac.uk)

# Introduction

Our study uses the shared socio-economic pathways projected in the IMAGE 3.2 database coupled to the GDPPS model of soil phosphorus to predict the amount of phosphate rock required to sustain Sub-Saharan African agriculture from 2020 to 2050. This document contains additional detail on the shared socio-economic pathways, crop-production in IMAGE 3.2, the GDPPS model and the soil hysteretic process.

# Shared Socio-Economic Pathways

Shared Socio-Economic pathways were developed in 2014 by O’Neil et al. ^1^ and van Vuuren et al. ^2^ to provide a framework for human behaviour within climate modelling to 2100 and by 2017 each pathway had a descriptive narrative ^3^. These pathways were built into the IMAGE 3.2 database for environmental modelling ^4^. We reproduce these narratives in Table *1* ^[[1]](#footnote-2)^ to provide additional context for our findings.

Table 1 Shared Socio-Economic pathways narratives reproduced (with edits) from O’Neil et al. ^3^ under a licence agreement *.

| **SSP1: Sustainability—Taking the green road**  The world shifts gradually, but pervasively, toward a more sustainable path, emphasizing more inclusive development that respects perceived environmental boundaries. […] The emphasis on economic growth shifts toward a broader emphasis on human well-being, even at the expense of somewhat slower economic growth over the longer term. Driven by an increasing commitment to achieving development goals, inequality is reduced both across and within countries. Investment in environmental technology and changes in tax structures lead to improved resource efficiency, reducing overall resource use and improving environmental conditions over the longer term. […] Consumption is oriented toward low material growth and lower resource intensity. Directed development of environmentally friendly technologies [and] institutions that can facilitate international cooperation results in relatively **low challenges to mitigation**. At the same time, the improvements in human well-being, along with strong and flexible global, regional, and national institutions imply **low challenges to adaptation**. |
| --- |
| **SSP2: Middle of the road**  The world follows a path in which social, economic, and technological trends do not shift markedly from historical patterns. Development and income growth proceeds unevenly, with some countries making relatively good progress while others fall short of expectations. Most economies are politically stable. Globally connected markets function imperfectly. Global and national institutions work toward but make slow progress in achieving sustainable development goals, including improved living conditions and access to education, safe water, and health care. Technological development proceeds apace, but without fundamental breakthroughs. Environmental systems experience degradation, although there are some improvements and overall the intensity of resource use declines. […] Global population growth is moderate […], however, education investments are not high enough to accelerate the transition to low fertility rates in low-income countries and to rapidly slow population growth. This growth, along with income inequality that persists or improves only slowly, continuing societal stratification, and limited social cohesion, maintain challenges to reducing vulnerability to societal and environmental changes and constrain significant advances in sustainable development. These moderate development trends leave the world, on average, facing **moderate challenges to mitigation and adaptation**, but with significant heterogeneities across and within countries. |
| **SSP3: Regional rivalry—A rocky road**  A resurgent nationalism, concerns about competitiveness and security, and regional conflicts push countries to increasingly focus on domestic or, at most, regional issues. This trend is reinforced by the limited number of comparatively weak global institutions, with uneven coordination and cooperation for addressing environmental and other global concerns. Policies shift over time to become increasingly oriented toward national and regional security issues, including barriers to trade, particularly in agricultural markets. Countries focus on achieving food security goals within their own regions at the expense of broader-based development […]. Investments in education and technological development decline. Economic development is slow, consumption is material-intensive, and inequalities persist or worsen over time, especially in developing countries. There are pockets of extreme poverty alongside pockets of moderate wealth, with many countries struggling to maintain living standards and provide access to safe water, improved sanitation, and health care for disadvantaged populations. A low international priority for addressing environmental concerns leads to strong environmental degradation in some regions. The combination of impeded development and limited environmental concern results in poor progress toward sustainability. Population growth is high in developing countries. Growing resource intensity along with difficulty in achieving international cooperation and slow technological change imply **high challenges to mitigation**. The limited progress on human development, slow income growth, and lack of effective institutions, especially those that can act across regions, implies **high challenges to adaptation** for many groups in all regions. |
| **SSP4: Inequality—A road divided**  Highly unequal investments in human capital, combined with increasing disparities in economic opportunity and political power, lead to increasing inequalities and stratification both across and within countries. Over time, a gap widens between an internationally-connected society that is well educated and contributes to knowledge- and capital-intensive sectors of the global economy, and a fragmented collection of lower-income, poorly educated societies that work in a labour intensive, low-tech economy. Power becomes more concentrated in a relatively small political and business elite, even in democratic societies, while vulnerable groups have little representation in national and global institutions. Economic growth [in] low-income countries [is low and many struggle] to provide adequate access to water, sanitation and health care for the poor. [There is a] well-integrated international political and business class capable of acting quickly and decisively, implies **low challenges to mitigation**. **Challenges to adaptation are high** for the substantial proportions of populations at low levels of development and with limited access to effective institutions for coping with economic or environmental stresses. |
| **SSP5: Fossil-fuelled development—Taking the highway**  Driven by the economic success of industrialized and emerging economies, this world places increasing faith in competitive markets, innovation and participatory societies to produce rapid technological progress and development of human capital as the path to sustainable development. Global markets are increasingly integrated, with interventions focused on maintaining competition and removing institutional barriers to the participation of disadvantaged population groups. There are also strong investments in health, education, and institutions to enhance human and social capital. At the same time, the push for economic and social development is coupled with the […] adoption of resource intensive lifestyles around the world. All these factors lead to rapid growth of the global economy. There is faith in the ability to effectively manage social and ecological systems, including by geo-engineering if necessary. While local environmental impacts are addressed effectively by technological solutions, there is relatively little effort to avoid potential global environmental impacts due to a perceived trade-off with progress on economic development. Global population peaks and declines in the 21st century [because] fertility declines rapidly in developing countries […]. International mobility is increased by gradually opening up labour markets as income disparities decrease. The lack of global environmental concern result in potentially **high challenges to mitigation**. The attainment of human development goals, robust economic growth, and highly engineered infrastructure results in relatively **low challenges to adaptation** to any potential climate change for all but a few. |

# Crop-production in IMAGE 3.2

The different shared socio-economic pathways within IMAGE 3.2 have contrasting total crop-productions which we discuss in the main text. However, the model has 16 different crop types within it: Maize, Oil – palm fruit, other non-food (e.g. luxury, spices), other temperature cereals, plant based fibres, pulses, rice, soy beans, sugar crops, temperate oilcrops, temperate roots and tubers, tropical cereals, tropical oilcrops, tropical roots and tubers, vegetables and fruits, and wheat. These follow subtly different pathways and by 2050 IMAGE 3.2 projects only small variations between the crop ratios for the different pathways but three crop types, tropical roots and tubers, maize, and tropical cereals dominate Sub-Saharan Africa crop production making up 29 ± 1 %, 14 ± 1 % and 10 ± 1 %, respectively, with all other crops consisting of the other 48 ± 2 %.


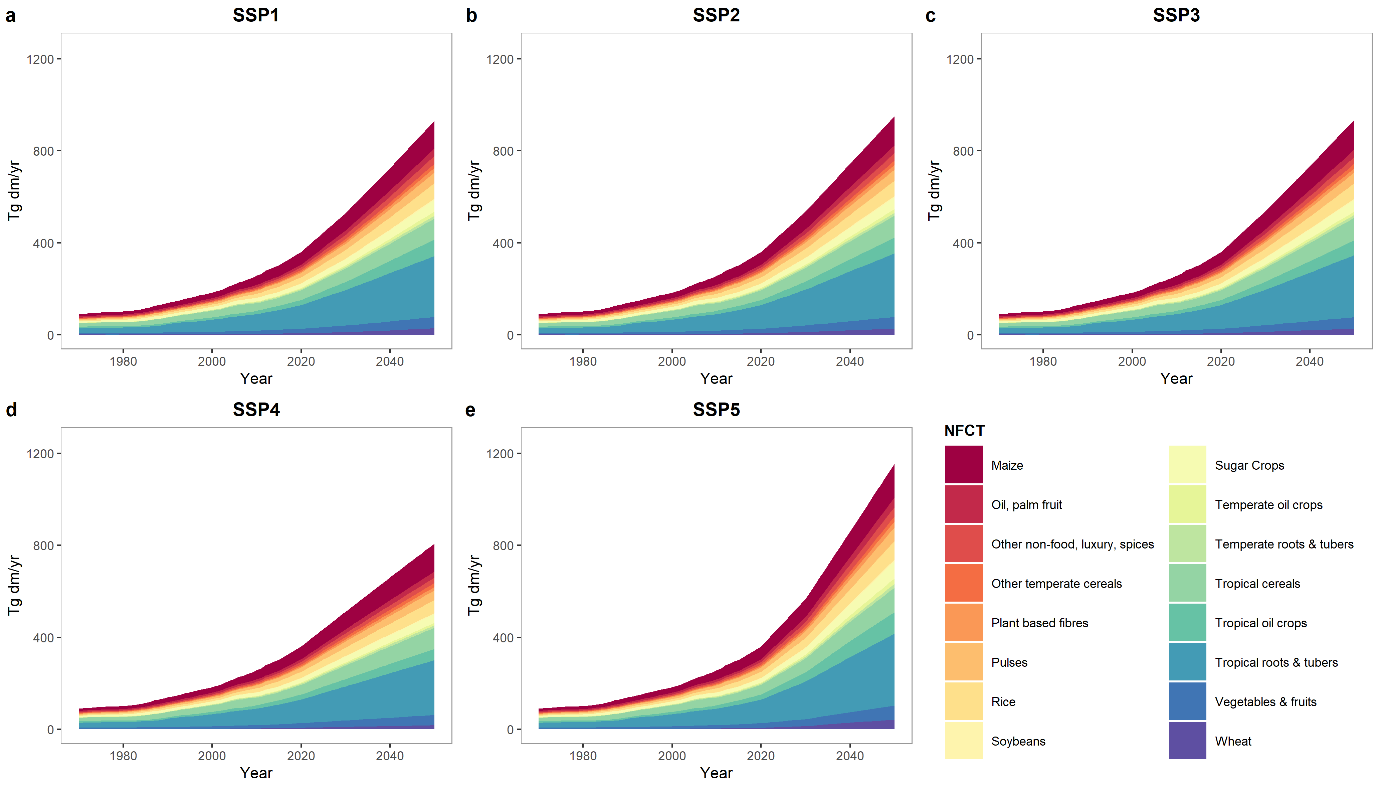


Figure 1 IMAGE 3.2 crop production predictions for SSP1 – 5 divided into different crop types.

# GDPPS model description

Within GDPPS soil phosphorus can exist in multiple forms, called pools, for the purposes of this research (and in keeping with earlier modelling studies) two pools are considered; labile and stable ^5–8^. The labile pool is that from which plants can uptake phosphorus, in particular from a sub-pool called available P ^9^, whilst the stable pool is inaccessible to plants (Figure 2).

Most fertilizers and manures are input to the labile pool however phosphorus can transfer between these two pools (Figure 2). The transfer from labile to stable pool is controlled by the concentration of weathered (oxalate) Fe and Al: the more oxalate Fe and Al the greater the transfer rate ^7,8,10,11^. This means that the concentration of weathered Fe and Al largely controls the efficiency of phosphorus use because if transfer from labile to stable is high then efficiency is low. Oxalate Al and Fe concentrations will vary spatially both within and between soil types.


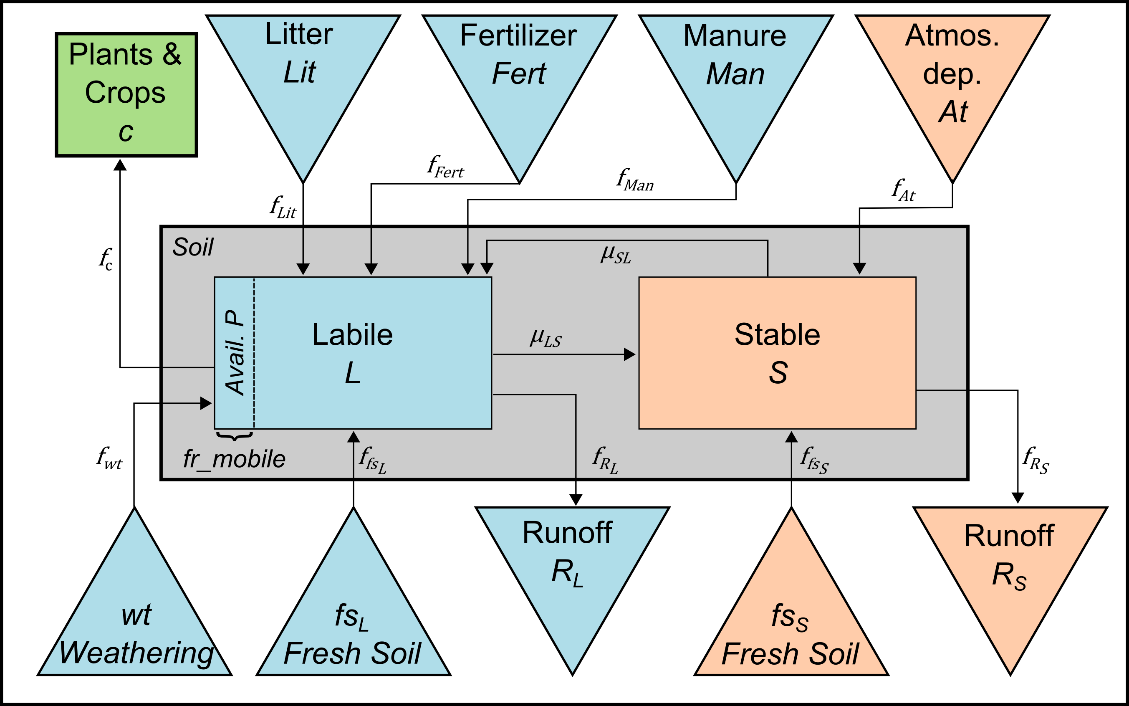


Figure 2 Sketch of GDPPS reproduced from Magnone et al., ^8^ under a CC BY 4.0 licence.

Previous studies of Western Europe ^6,12^ and Japan ^13^ show that crop yields continue to increase decades after high rates of application have ceased – at a continental scale this has not happened in Africa. However, in this study, we have shown evidence of historical hysteresis in Nigeria, Central African Republic and Ethiopia but no other countries. Nevertheless, even in these countries, hysteresis is not expected to be enough to increase the yields required to sustain food security to 2050 and neither is it expected at a continental scale. To understand the location specific nature of this phenomena consideration must be given to processes within the soil.

If soil phosphorus is measured through time four distinct modes can occur in relation to the labile to stable pools (Figure 3): *A)* an “increasing mode” where sizes of both labile and stable pools increase, leading to soil P improvement, *B)* a “decreasing mode” where sizes of both labile and stable pools decrease, leading to soil P depreciation, *C)* “SP to LP transfer mode” where stable pool size gets smaller whilst the labile pool size gets larger, *D)* “LP to SP transfer mode” where stable pool size gets larger whilst the labile pool size gets smaller. Soil P in modes C and D can either increase, decrease or be constant with time. These four modes are visualised in Figure 3 and the slopes of lines represent the readiness and availability of P to the plant (via the labile pool) in different soils*.* For example, in Figure 3A the steeper the angle of the line, the less responsive soil is because when the slope is greater more P is sequestrated in the stable pool.

In this supplementary information, we explain the soil hysteric process at a soil-scale level for each country.


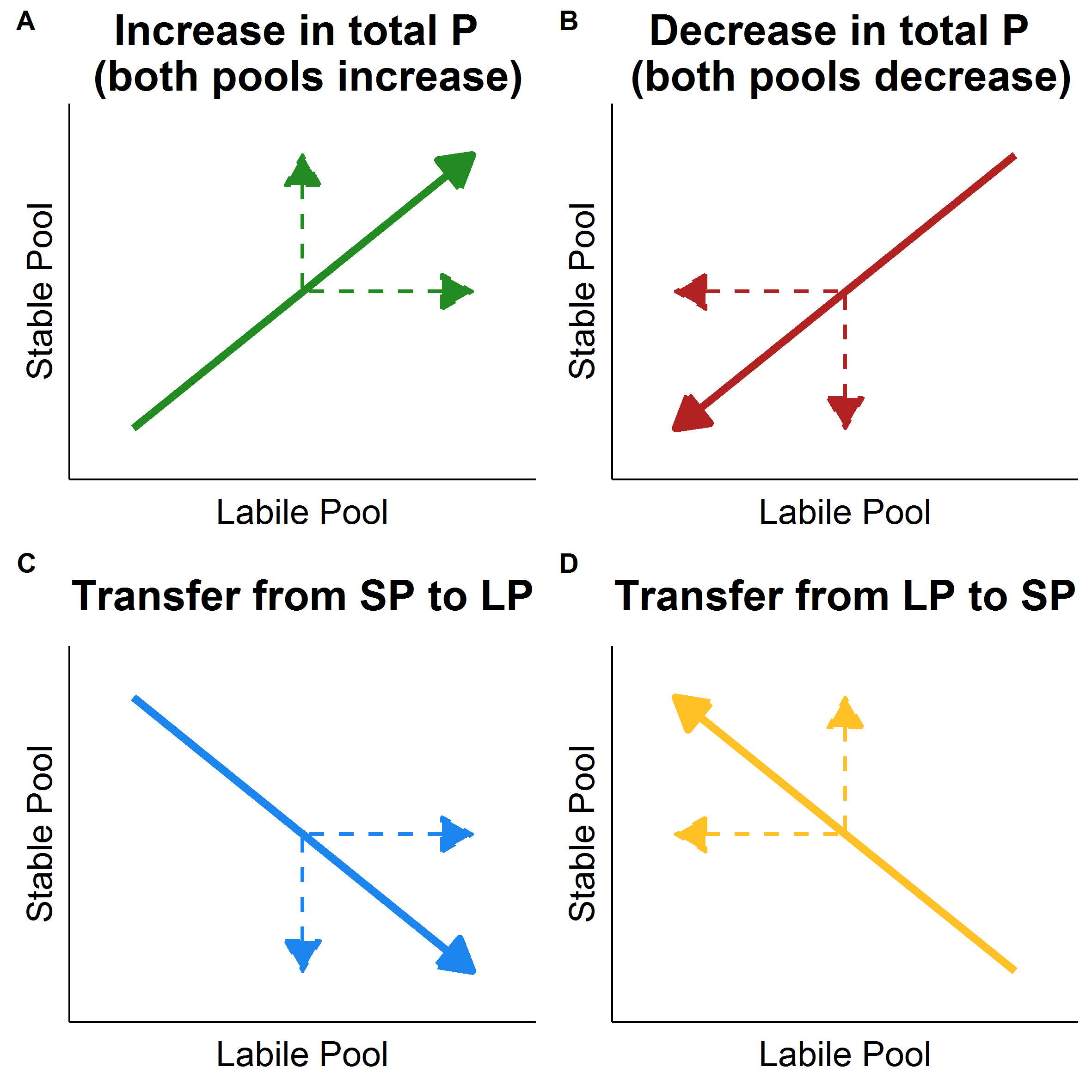


Figure 3 Different soil P modes. Increasing mode (A), decreasing mode (B), SP-LP transfer mode (C) and LP-SP transfer mode (D). The direction of the arrow represents time.

# Additional method information – base datasets for GDPPS

We calculated the concentration of oxalate Fe and Al , referred to as M [kg ha^-1^] in Equation 2, using the 250 m gridded continental extractable Fe and Al concentration raster maps produced by Hengl *et al.* as part of the ISRIC SoilGrids project ^14^. These maps were produced using source material of soil samples from about 59,000 locations combined with remote sensing co-variates to cover the whole continent; these samples were collected between 1938 and 2011 ^15^. Our model requires an assumption of a stable M during the modelling period. To check the validity of this assumption we conducted an analysis of variance (ANOVA) on the concentration of M from the source data. We first checked for year-on-year changes and then for decade-on-decade changes.

# Results and discussion

## Hysteresis Analysis

In the main text we demonstrated that four countries have exhibited hysteresis historically: Nigeria: between 1990 to 2000, South Africa between 1980 and 2010, D. R. Congo between 1980 and 2010, and Cameroon between 1980 and 2010.

Our data show that in all cases where hysteresis occurred a replenishment of the labile pool from the stable pool by the SP to LP transfer mode occurred (Figure 3). Nigeria’s hysteresis corresponds with a period of declining stable pool and stationary labile pool which occurred from 1970 to 2020 (Figure 4A). South Africa’s hysteresis corresponds with a period of constant stable pool and increasing labile pool from 1970 to 2005 (Figure 4C). D. R. Congo’s hysteresis corresponds with a slight decline in the stable pool and large increase in the labile pool between 1970 and 2000 (Figure 4E). Finally, Cameroon’s hysteresis corresponds with a period of constant stable pool and increasing labile pool between 1970 and 2015 (Figure 4G). This means that these soils have a preference for phosphorus to be in the labile pool over the stable pool.

Of the nations that did not exhibit hysteresis Ethiopia, Benin and Mali have all seen an increase in both pools (Figure 4B, I and J) whilst Ghana, Coté d’ Ivoire and Tanzania have seen decreases in both pools (Figure 4D, F and H). We project that all nations in the coming decades, under SSP2, will have increases in both pools, regardless of their phosphorus history and it is for this reason that no hysteresis is predicted.


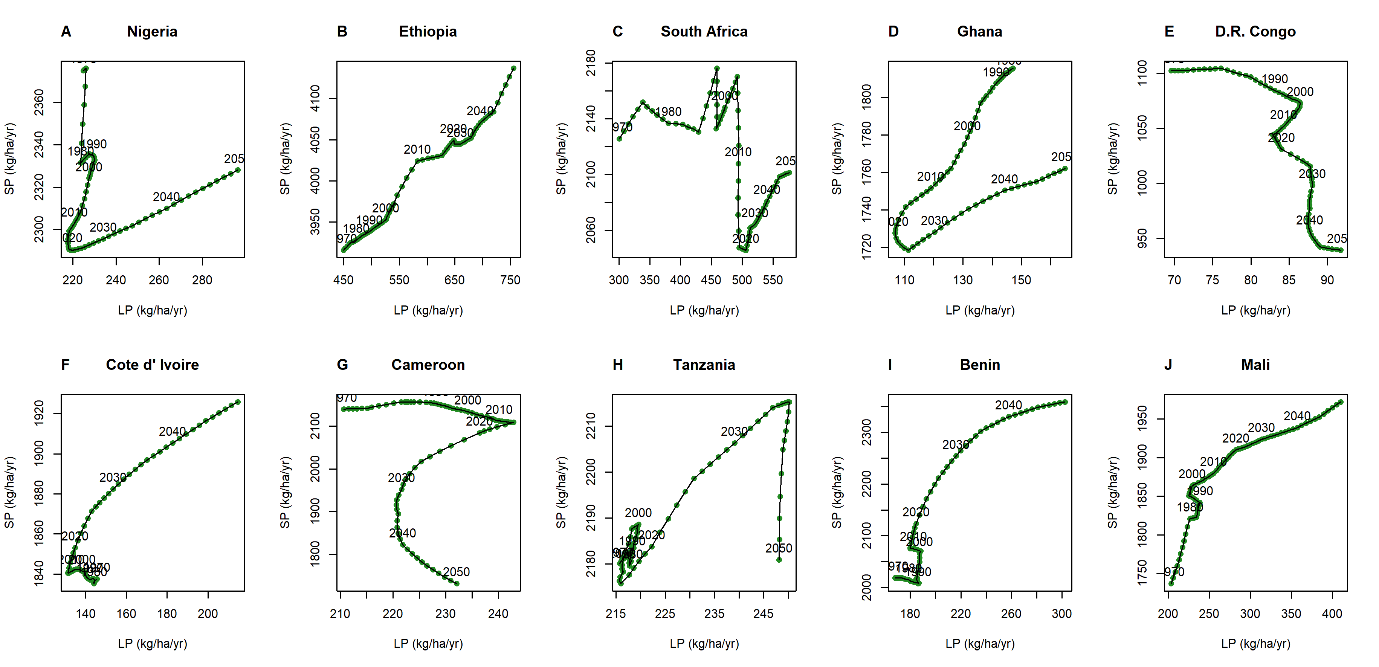


Figure 4 Changes in the labile and stable pools between 1970 and 2020 for the 10 countries expected to have the highest amounts of phosphorus use to 2050.

## Stability of soil oxalate Fe and Al (M)

Our ANOVA results indicate that the concentration of oxalate Fe and Al (referred to as M in Equation 2) does vary both between years (F[47,4214] = 8.84, p <0.01) and between decades (F[5,4252]=14.14, p <0.01). However, the post hoc Krustal-Wallis test, as a Dunn test, indicates that there is no significant difference in the mean M concentrations for the decades of the 1950s, 1970s, 1990s and 2000s and it is only the 1960s and 1980s which have significant differences in M. Due to the scale of data collection, this data was not collected with repeat samples and locations vary over time and may account for some of the variations observed. Thus, we believe that an assumption of a stable M throughout the model period is appropriate in this case.

# Conclusions

These results have demonstrated that it is the intrinsic soil properties which control the efficiency of phosphorus use. This efficiency is controlled by pool transfer processes which in turn are controlled by the iron and aluminium oxide concentrations in the soil.

# References

1. O’Neill, B. C. *et al.* A new scenario framework for climate change research: the concept of shared socioeconomic pathways. *Clim. Change* **122**, 387–400 (2014).

2. van Vuuren, D. P. *et al.* A new scenario framework for Climate Change Research: Scenario matrix architecture. *Clim. Change* **122**, 373–386 (2014).

3. O’Neill, B. C. *et al.* The roads ahead: Narratives for shared socioeconomic pathways describing world futures in the 21st century. *Glob. Environ. Chang.* **42**, 169–180 (2017).

4. Stehfest, E. *et al.* *Integrated Assessment of Global Environmental Change with IMAGE 3.0. Model description and policy applications*. (PBL Netherlands Environmental Assessment Agency, The Hague, 2014).

5. Zhang, J. *et al.* Spatiotemporal dynamics of soil phosphorus and crop uptake in global cropland during the 20th century. *Biogeosciences* **14**, 2055–2068 (2017).

6. Sattari, S., Bouwman, A. F., Giller, K. E. & van Ittersum, M. K. Residual soil phosphorus as the missing piece in the global phosphorus crisis puzzle. *Proc. Natl. Acad. Sci. U. S. A.* **109**, 6348–53 (2012).

7. Magnone, D. *et al.* Efficiency of phosphorus resource use in Africa as defined by soil chemistry and the impact on crop production. *Energy Procedia* **123**, 97–104 (2017).

8. Magnone, D. *et al.* Soil Chemistry Aspects of Predicting Future Phosphorus Requirements in Sub-Saharan Africa. *J. Adv. Model. Earth Syst.* **11**, 327–337 (2019).

9. Olsen, S. R., Cole, C. V., Watanabe, F. S. & Dean, L. A. Estimation of available phosphorus in soils by extraction with sodium bicarbonate. *USDA Circ.* **939**, 1–19 (1954).

10. Freese, D., van der Zee, S. E. A. T. M. & van Riemsdijk, W. H. Comparison of different models for phosphate sorption as a function of the iron and aluminium oxides of soils. *J. Soil Sci.* **43**, 729–738 (1992).

11. van der Zee, S. E. A. T. M. & van Riemsdijk, W. H. Model for Long-term Phosphate Reaction Kinetics in Soil. *J. Environ. Quali.* **17**, 35–41 (1988).

12. Verloop, J., Oenema, J., Burgers, S. L. G., Aarts, H. F. M. & van Keulen, H. P-equilibrium fertilization in an intensive dairy farming system: effects on soil-P status, crop yield and P leaching. *Nutr. Cycl. Agroecosystems* **87**, 369–382 (2010).

13. Mishima, S., Endo, A. & Kohyama, K. Recent trends in phosphate balance nationally and by region in Japan. *Nutr. Cycl. Agroecosystems* **86**, 69–77 (2010).

14. Hengl, T. *et al.* Soil nutrient maps of Sub-Saharan Africa: assessment of soil nutrient content at 250 m spatial resolution using machine learning. *Nutr. Cycl. Agroecosystems* **109**, 77–102 (2017).

15. Leenaars, J. G. B., Van Oostrum, A. J. M. & Ruiperez Gonzalez, M. *Africa Soil Profiles Database Version 1.2 A compilation of georeferenced and standardised legacy soil profile data for Sub-Saharan Africa (with dataset)*. (2014).

1. Reprinted from Global Environmental Change, 42, O’Neill, B.C., Kriegler, E., Ebi, K.L., Kemp-Benedict, E., Riahi, K., Rothman, D.S., van Ruijven, B.J., van Vuuren, D.P., Birkmann, J., Kok, K. and Levy, M., The roads ahead: Narratives for shared socioeconomic pathways describing world futures in the 21st century, Pages No. 169-180, Copyright (2017), with permission from Elsevier. [↑](#footnote-ref-2)
